# Editorial: Chemoinformatics Approaches to Structure- and Ligand-Based Drug Design

**DOI:** 10.3389/fphar.2018.01416

**Published:** 2018-12-04

**Authors:** Leonardo L. G. Ferreira, Adriano D. Andricopulo

**Affiliations:** Laboratory of Medicinal and Computational Chemistry, Center for Research and Innovation in Biodiversity and Drug Discovery, Physics Institute of Sao Carlos University of Sao Paulo, Sao Carlos, Brazil

**Keywords:** drug design, molecular modeling, computational chemistry, QSAR, molecular docking, QSPR, virtual screening

Pharmaceutical research and development (R&D) has faced outstanding challenges as scientific breakthroughs achieved in the past two decades have revolutionized the field. Important approaches such as high-throughput screening (HTS) have increasingly been used in combination with emerging strategies relying on genomics, chemical biology and molecular modeling (Jones and Bunnage, 2017). These forefront approaches have promoted substantial progress in our understanding of key biological processes, in addition to fostering critical advances in the armamentarium available for drug R&D (Liu et al., 2017). Along with synthetic strategies such as combinatorial chemistry, which has supported a consistent expansion of the chemical space explored in drug discovery, these state-of-the-art technologies are shaping the future of pharmaceutical industry. The integration of these methodologies to the drug discovery enterprise has led to an exponential growth of chemical and biological data, in addition to a sharp increase in the complexity of the R&D process. As a result, current players in drug discovery have invested unprecedentedly in the development of computational methods to extract meaning from these data and simulate critical phenomena related to drug efficacy, pharmacokinetics (PK) and toxicity (Macalino et al., 2015). The value of using *in silico* strategies has been demonstrated by the increasing number of publications reporting campaigns that have resulted in the discovery of promising lead compounds; many of them undergoing clinical development and reaching the market.

Usually, these computer-assisted efforts integrate ligand- and structure-based drug design strategies (LBDD and SBDD, respectively) with a combination of experimental techniques (Ferreira et al., 2015). Broadly used SBDD approaches, molecular docking, homology modeling, molecular dynamics and structure-based virtual screening have provided relevant insights into ligand-receptor interactions (Wang et al., 2016). Equally important, LBDD methods such as pharmacophore modeling, quantitative structure-activity relationships (QSAR) and ligand-based virtual screening have been actively used to explore small-molecule databases and produce correlations between chemical features and pharmacological activity (Lavecchia, 2015). Also a hot-topic in LBDD, quantitative structure-property relationship (QSPR) models are central for predicting PK and toxicity-related characteristics (Tao et al., 2015).

Including original and review articles, this research topic (RT) connects recent applications of LBDD and SBDD with the study of drug activity, as well as drug absorption, distribution, metabolism, excretion (ADME) and toxicity. We were able to collect 31 articles connecting more than 200 scientists from all around the world. Molecular modeling strategies for different conditions such as cancer, leishmaniasis, malaria, coronary heart disease, diabetes, and Alzheimer's disease are addressed. A broad variety of topics including the development of scoring functions, nuclear magnetic resonance (NMR)-assisted molecular docking, and the interplay between molecular docking and molecular dynamics are covered. In addition, this RT highlights the use of natural products as inhibitors of molecular targets such as the epidermal growth factor receptor (EGFR) and tumor necrosis factors (TNFs). New ligands targeting protein arginine methyltransferases, Kirsten rat sarcoma viral oncogene homolog (KRAS), G protein-coupled receptors (GPCRs), matrix metalloproteinases, heat shock proteins (HSPs), and mammalian Disheveled, are also considered. The design and implementation of online platforms for the prediction of *in vivo* toxicity, off-target interactions, and PK properties are described. The use of chemical proteomic approaches to profile molecular targets, force-fields for accessing compound-solvent interactions, and algorithms that consider synthetic accessibility for lead optimization are also reported.

It is our aim that the high-quality material enclosed in this RT contributes to the dissemination of outstanding science across the worldwide research community dedicated to the fascinating universe of drug discovery.

## Author Contributions

All authors listed have made a substantial, direct and intellectual contribution to the work, and approved it for publication.

### Conflict of Interest Statement

The authors declare that the research was conducted in the absence of any commercial or financial relationships that could be construed as a potential conflict of interest.
